# Supplementary material for: Causes and consequences of sex-chromosome turnovers in Diptera
Source: Evol Lett. 2026 Mar 12;10(3):253–68. doi: 10.1093/evlett/qrag003 (PMC13231274; doi:10.1093/evlett/qrag003)
Supplement: qrag003_Supplemental_Files [file qrag003_supplemental_files.zip › Supplementary_Datasets.zip!/Supplementary_Datasets/Dataset_2_Graphical_representation_outgroups/Drosophilidae_Drosophila_virilis_chromosomes.pdf]

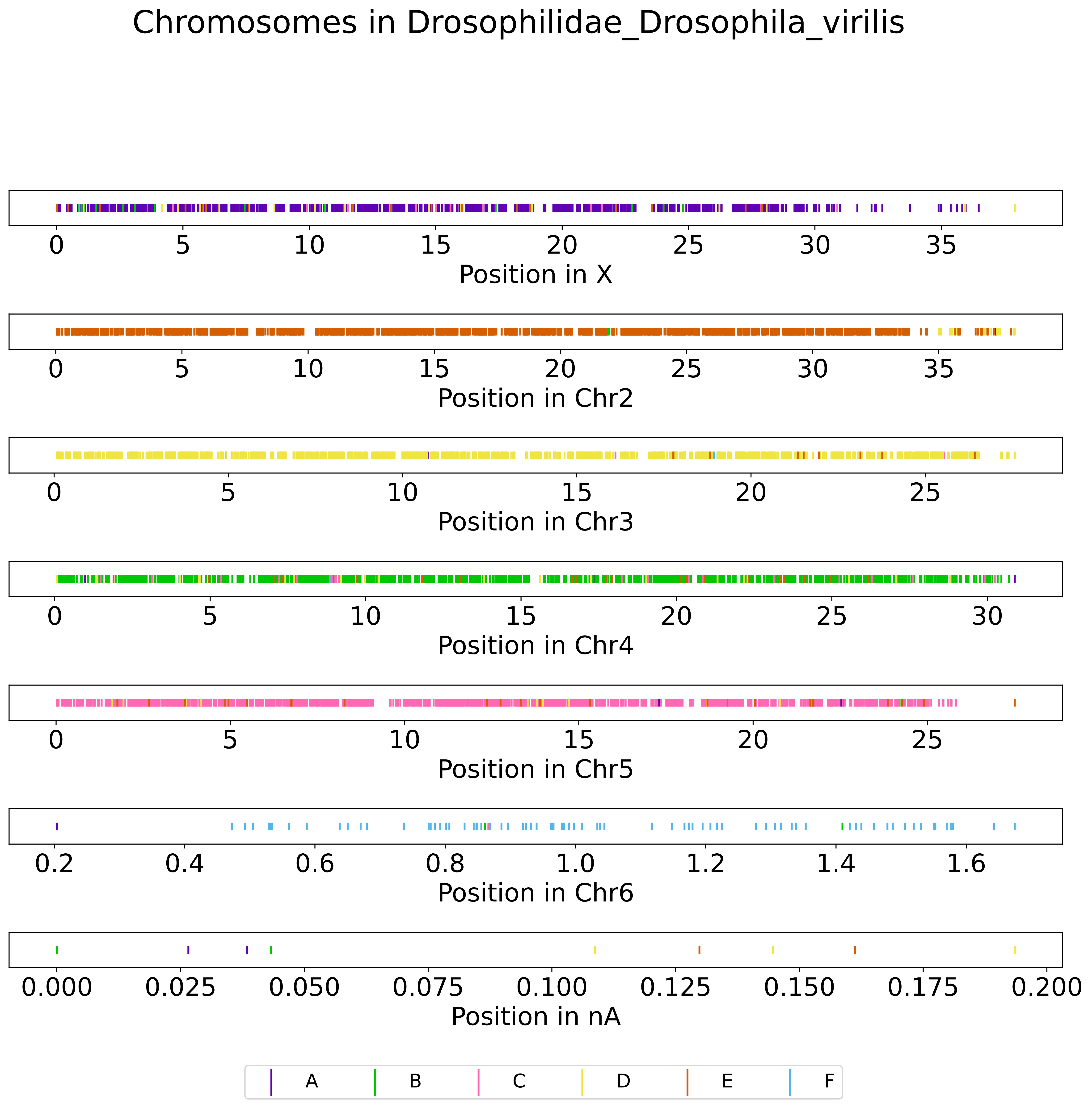
Chromosomes in Drosophilidae_Drosophila_virilis
0
5
10
15
20
25
30
35
Position in X
Position in Chr2
Position in Chr3
Position in Chr4
Position in Chr5
0.2
0.4
0.6
0.8
1.0
1.2
1.4
1.6
Position in Chr6
0.000
0.025
0.050
0.075
0.100
0.125
0.150
0.175
0.200
Position in nA
A
B
C
D
E
F
